# Efficacy and safety of FOLFIRI/aflibercept in second‐line treatment of metastatic colorectal cancer in a real‐world population: Prognostic and predictive markers

**DOI:** 10.1002/cam4.1903

**Published:** 2019-01-28

**Authors:** Ana Fernández Montes, Nieves Martínez Lago, Marta Covela Rúa, Juan de la Cámara Gómez, Paula González Villaroel, José Carlos Méndez Méndez, Mónica Jorge Fernández, Mercedes Salgado Fernández, Margarita Reboredo López, Guillermo Quintero Aldana, María Luz Pellón Augusto, Begoña Graña Suárez, Jesús García Gómez

**Affiliations:** ^1^ University Hospital of Ourense Ourense Spain; ^2^ University Hospital of A Coruña A Coruña Spain; ^3^ Universital Hospital Lucus Augusti Lugo Spain; ^4^ University Hospital Arquitecto Marcide Ferrol Spain; ^5^ Oncological Center of Galicia A Coruña Spain; ^6^ Univerity Hospital Álvaro Cunqueiro Vigo Spain

**Keywords:** aflibercept, FOLFIRI, metastatic colorectal cancer, real‐life study, routine clinical practice, VELOUR

## Abstract

**Purpose:**

The phase III VELOUR trial demonstrated efficacy with combined FOLFIRI‐aflibercept in patients with metastatic colorectal cancer previously treated with oxaliplatin with or without bevacizumab versus placebo. The effect of FOLFIRI‐aflibercept in routine clinical practice was evaluated.

**Methods/Patients:**

Overall survival (OS), progression‐free survival (PFS), response and safety were analysed for 78 patients treated with FOLFIRI‐aflibercept at six GITuD institutions. Exploratory analyses of prognostic and predictive markers of efficacy were performed.

**Results:**

Patients had good general status (PS 0‐1 96.2%), tumours were mostly *RAS*‐mutant (75.6%), synchronous (71.8%), and left‐sided (71.8%). Prior therapy included bevacizumab (47.4%) and anti‐EGFR agents (12.8%). PFS was longer for metachronous than synchronous tumours (11.0 vs 5.0 months, *P* = 0.028), and for left‐colon tumours (7.0 vs 3.0 months, *P* = 0.044). *RAS‐*mutant status, first‐line treatment and primary tumour surgery did not impact PFS. The disease control rate was 70.5%. The most common grade 3/4 toxicities were neutropenia (15.3%), asthenia (10.3%), diarrhea and mucositis (6.4% each). Dysphonia was reported in 39.7% of patients, and grade 3 hypertension in 3.8%. Development of hypertension (any grade) was significantly associated with a reduced risk of progression by multivariate analysis (HR = 2.7; 95%CI 1.3‐5.4; *P* = 0.001).

**Conclusions:**

Efficacy with FOLFIRI‐aflibercept in a real‐life population was in line with results from the pivotal trial and toxicity was manageable with treatment adaptation. Survival outcomes were not impacted by primary tumour location, *RAS*‐mutant status, first‐line treatment or primary tumour surgery. Hypertension may be a surrogate marker of efficacy in this patient population.

## 1 INTRODUCTION

Colorectal cancer is the second most common cancer in Europe and a primary cause of death worldwide.^1^ The backbone of first‐line treatment of metastatic colorectal cancer (mCRC) relies on a combination of a fluoropyrimidine (FU), leucovorin and either oxaliplatin (FOLFOX) or irinotecan (FOLFIRI). Today, the vast majority of patients with mCRC are also treated with a biological agent in the first‐line setting, typically monoclonal antibodies against vascular endothelial growth factor (VEGF) or epidermal growth factor receptor (EGFR), depending on their *RAS* mutation status.^2^

Aflibercept is one such agent, a recombinant anti‐angiogenic fusion protein, which selectively blocks the A and B isoforms of vascular endothelial growth factor (VEGF) and placental growth factor (PIGF),^3^ differing from bevacizumab, which selectively blocks only VEGF‐A. The benefit of the addition of aflibercept to FOLFIRI compared to placebo with FOLFIRI in patients with mCRC who had progressed on oxaliplatin‐based chemotherapy, was confirmed in the phase III multicentre randomised VELOUR trial,^4^ with improved median overall survival (OS) of 13.5 vs 12.1 months respectively (hazard ratio [HR] 0.82, 95% CI, 0.71‐0.94, *P* = 0.003). Benefit was also reported in terms of progression‐free survival (PFS) at 6.9 vs 4.7 months (HR 0.76 95% CI, 0.66‐0.87), and overall response rate (ORR) with a 9% increase in favor of the combination (19.8% vs 11.1%).

The extent of benefit was also demonstrated in all predetermined subgroups, including patients previously exposed to bevacizumab,^5^ although they represented only 30% of patients. The proportion of the population who had received an anti‐EGFR was however unknown. A post‐hoc multivariate analysis of the VELOUR study profiled various subgroups with a greater survival benefit^6^; patients with an ECOG performance status (PS) of 0 and any number of metastatic sites or with PS 1 and no more than one metastatic site presented an increase of 3.1 months in OS (16.2 vs 13.1 months). The prognostic role of *RAS* mutation status, tumour laterality, and the impact of first‐line treatment are acknowledged influences of outcome for treatment of mCRC with FOLFOX/FOLFIRI with or without cetuximab or bevacizumab,^7^, ^8^ however little is known in the context of patients treated with FOLFIRI‐aflibercept. Analysis of outcome according to mutational status of *KRAS*, *RAS* and *BRAF* from the VELOUR translational study was recently published.^9^ A total of 482 samples (39.3% of ITT population) were retrospectively analysed by next generation sequencing. Median OS was 16.0 months for aflibercept and 11.7 months for placebo in the *RAS* wild‐type population (HR 0.70 95% CI, 0.50‐0.97), and 12.6 months vs 11.2 months respectively in *RAS*‐mutant patients (HR 0.93 95% CI, 0.7‐1.23), which did not reach significance indicating that aflibercept is effective regardless of *RAS* status.

A retrospective study was conducted in a real‐world population of mCRC patients. It was designed to evaluate the impact of FOLFIRI‐aflibercept as second‐line treatment or following rapid progression while receiving oxaliplatin as adjuvant therapy on the extent of the benefit. Given the importance of surrogate efficacy markers for the FOLFIRI‐aflibercept combination as a clinical tool, we performed exploratory analyses to identify predictive and prognostic factors for survival outcomes.

## 2 MATERIALS AND METHODS

### 2.1 Study design

We conducted a retrospective, multicentre, observational study of patients with mCRC treated with FOLFIRI‐aflibercept after progression on an oxaliplatin‐based first‐line regimen, or after an interval of less than six months following oxaliplatin‐based adjuvant treatment (termed rapid progressors), as part of routine clinical practice at six hospitals from the Galician Research Group on Digestive Tumours (GITuD) network. The study was approved by a local ethics committee and was performed in accordance with the Declaration of Helsinki. All patients gave informed consent prior to inclusion.

To be eligible, patients had to have been treated with FOLFIRI‐aflibercept as part of routine clinical practice and the same criteria used in the VELOUR trial^4^ were applied. Of note, patients who had received prior irinotecan, any other anti‐angiogenic drugs, or aflibercept with chemotherapy other than FOLFIRI were excluded.

Patients received 4 mg/kg of aflibercept (intravenously [IV]), over 1 hour on day 1 every 2 weeks, followed immediately by the FOLFIRI regimen (irinotecan 180 mg/m^2^ IV over 90 minutes, with leucovorin 400 mg/m^2^ IV over 2 hours, followed by FU 400 mg/m^2 ^bolus and FU 2400 mg/m^2^ continuous infusion over 46 hours).

### 2.2 Data collection

Clinico‐pathological and treatment data were collected from clinical records, including sex, age, relevant medical and surgical history, vascular comorbidities, arterial or venous thromboembolic events, gastrointestinal perforation or fistula, PS, neutrophil to lymphocyte ratio (NLR), the presence of thrombocytosis, and heparin treatment prior to treatment start. Disease characteristics included *RAS* mutation status, primary tumour location, tumour presentation, primary tumour surgery, the number of metastatic locations and type of first‐line therapy. The number of FOLFIRI‐aflibercept cycles received and the number of lines received after progression on the FOLFIRI‐aflibercept were recorded, along with disease progression and survival status.

### 2.3 Statistical analyses

Overall survival was defined as the time between treatment start and death by any cause. PFS was defined as the time from treatment start and confirmed radiologic progression or death by any cause. The ORR was defined as the proportion of patients who achieved a partial or complete response, and the disease control rate was defined as the proportion of patients who achieved a complete or partial response or stable disease lasting at least six weeks after the start of treatment. Toxicity was reported as per NCI‐CTCAE v4.0.

Statistical analyses were carried out using SPPS v20.0 (IBM, Ourense, Spain). The chi‐squared test or Fisher's exact test (depending on the sample size) was used to compare clinical and demographic variables. The Kaplan‐Meier model was applied to estimate median PFS and OS and 95% confidence intervals (CI). Differences between survival curves were compared using the log‐rank test with a two‐sided significance level of 0.05.

## 3 RESULTS

Data were collected from 78 patients treated with FOLFIRI‐aflibercept between January 2013 and May 2016. Patient characteristics are summarised in Table 1. Median age was 65 years (range 37‐81), with 29.5% of patients 70 years or older. All patients had been treated with oxaliplatin in either the first‐line metastatic (79.5%) or adjuvant (20.5%) setting.

**Table 1:** Patient characteristics

	No.	% (N = 78)
Age		
Median (range) in years	65 (37‐81)	
≥70 y	22	29.5
Sex		
Female	29	37.2
Male	49	62.8
ECOG PS		
0	17	21.8
1	58	74.4
2	3	3.8
Primary tumor location		
Left colon	56	71.8
Right colon	22	28.2
Number of metastatic sites		
1	4	5.1
2	38	48.7
3	35	44.9
4	1	1.3
Liver metastasis	53	67.9
Tumor presentation		
Synchronous	56	71.8
Metachronous	22	28.2
Primary tumor surgery	33	42.3
Mutational status		
KRAS	52	66.7
NRAS	7	9.0
First‐line treatment		
FOLFOX	23	29.5
FOLFOX +anti‐EGFR	10	12.8
FOLFOX +bevacizumab	37	47.4
Adjuvant only (rapid progressors)	8	10.3
Previous thromboembolic event	28	35.9
Prophylactic low‐weight heparin	4	5.1
Thrombocytosis	4	5.1
NLR		
Median (range)	2.4 (0.82‐6.71)	
<3	61	78.2

NLR, neutrophil to lymphocyte ratio

Patients were followed‐up for a median of 11.5 months (range, 1‐41). Progression on FOLFIRI‐aflibercept was reported in 96.2% of patients and 83.3% had died at the cut‐off. Median PFS was 6.8 months (95% CI, 4.4‐9.2 months) and median OS was 12.0 months (95%CI, 9.4‐14.6 months). All 78 patients were evaluable for response; none had a complete response, 17 had a partial response, and 38 patients had stable disease, giving an ORR and disease control rate of 21.8% and 70.5% respectively. After progression, 39 patients (49.9%) received one or more further treatment lines.

### 3.1 Prognostic factors

**Figure 1:**
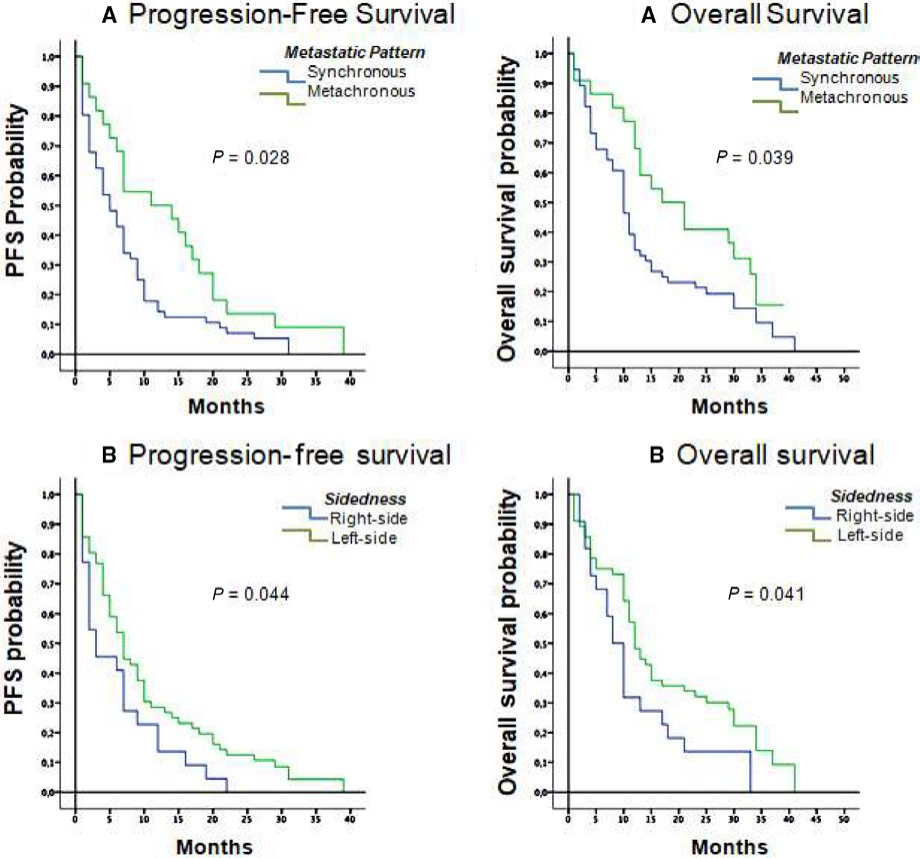
Kaplan‐Meier curves for progression‐free survival and overall survival according to metastasis pattern (A) and laterality (B)

Exploratory analyses to identify potential prognostic factors for PFS and OS were carried out (Table 2). Analysis of survival in patients who developed metachronous metastasis compared to synchronous metastasis revealed that the former group had significantly longer PFS (11.0 months, 95% CI, 4.1‐17.9) compared to patients with synchronous metastases (5.0 months, 95% CI, 3.0‐7.0; *P* = 0.028). The same pattern was observed for OS, with 17.0 months (95% CI, 7.8‐26.2 months) in metachronous versus 10.0 months (95% CI, 8.2‐11.8 months) in synchronous patients (*P* = 0.039; Figure 1A). Evaluation of tumour laterality showed that PFS was significantly longer in patients with left‐colon tumours, with a median of 7.0 months (95% CI, 5.2‐8.8 months) compared with 3.0 months (95% CI, 0.1‐5.9 months) in patients with right‐colon tumours (*P* = 0.044). The same trend was observed for OS, with a median of 12.0 months (95% CI, 9.9‐14.9 months) with the left colon versus 8.0 months (CI 95%, 5.70‐10.3 months) for the right colon (*P* = 0.041; Figure 1B).

**Table 2:** Progression‐free and overall survival according to prognostic factors

	Median PFS (95% CI) in months	*P*‐value	Median OS (95% CI) in months	*P*‐value
Metastases				
Metachronous	11.0 (4.1‐17.9)	0.028	17.0 (7.8‐26.2)	0.039
Synchronous	5.0 (3.0‐7.0)		10.0 (8.2‐11.8)	
Tumor laterality				
Left	7.0 (5.2‐8.8)	0.044	12.0 (9.9‐14.9)	0.041
Right	3.0 (0.1‐5.9)		8.0 (5.70‐10.3)	
RAS status				
Wild‐type	4.9 (0.9‐8.9)	1.0	11.9 (7.6‐16.4)	0.24
Mutant (KRAS/NRAS)	7.5 (6.4‐8.7)		12.3 (9.1‐15.5)	
ECOG				
0	9.9 (7.0‐12.8)	0.20	13.8 (9.7‐17.9)	0.30
1	5.3 (2.8‐7.9)		11.3 (8.3‐14.3)	
2	1.4 (1.3‐1.4)		1.6 (1.5‐1.7)	
Primary surgery				
Yes	7.0 (5.9‐8.3)	0.68	12.9 (9.5‐16.4)	0.73
No	5.5 (0.9‐10.2)		11.3 (9.7‐12.9)	

All other factors analyses, *RAS* mutation status, PS and surgery of the primary tumour were not significantly associated with the extent of survival benefit (Table 2).

### 3.2 Outcome predictive factors

**Figure 2:**
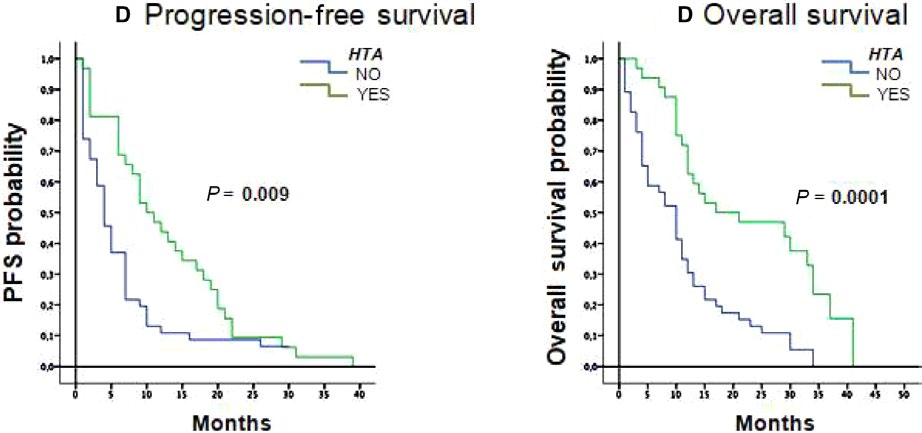
Kaplan‐Meier curves for progression‐free survival and overall survival according to the presence of hypertension (HTA)

Exploratory analyses to identify potential predictive factors for survival with FOLFIRI‐aflibercept were carried out (Table 3). Type of first‐line therapy (FOLFOX along vs bevacizumab vs anti‐EGFR) and early vs late progression did not have a significant association with survival. The presence of hypertension developing during treatment with FOLFORI‐aflibercept was however associated with a survival benefit, reducing the risk of progression, with significantly longer median PFS in patients who developed hypertension 10.6 months (95% CI, 6.3‐13.7) compared to 4.0 months (95% CI, 2.7‐5.3) in patients who did not develop hypertension (*P* = 0.009), with a hazard ratio of 2.7 (95%CI 1.3‐5.4; *P* = 0.001). OS was also significantly longer at 17.0 months (95% CI, 0‐35.5 months) for patients who developed hypertension versus 10.0 months (95% CI, 7.2‐12.8 months; *P* < 0.001; Figure 2). No interactions were identified between the presence of any other toxicities and survival.

**Table 3:** Progression‐free and overall survival according to predictive factors

	Median PFS (95% CI) in months	*P*‐value	Median OS (95% CI) in months	*P*‐value
First‐line treatment				
FOLFOX	6.7 (0‐14.1)	0.37	12.0 (5.2‐18.7)	0.36
FOLFOX/anti‐EGFR	4.9 (0.5‐9.3)		11.9 (1.0‐22.8)	
FOLFOX/bevacizumab	6.8 (4.1‐9.5)		10.8 (9.7‐11.8)	
Progression after adjuvant^a^				
Early	7.9 (3.5‐12.3)	0.49	15.2 (11.6‐18.8)	0.34
Late	6.7 (4.7‐8.6)		11.9 (10.3‐13.6)	
Hypertension				
Present	10.6 (6.3‐13.7)	0.009	17.0 (0‐35.5)	<0.001
Absent	4.0 (2.7‐5.3)		10.0 (7.2‐12.8)	

a Within 6 mo of completing adjuvant treatment

### 3.3 Safety

At the time of the analysis, patients had received a median of 10 two‐week cycles of FOLFIRI‐aflibercept (range 1‐49), with 11.5% receiving at least 25 cycles. Treatment delays occurred in 50 patients (64.1%) and 40 patients (51.3%) required dose reductions. The most common adverse events occurring during treatment with FOLFIRI‐aflibercept are summarised in Table 4. They included asthenia (85% of patients), diarrhea (64%), mucositis (58%), neutropenia (46%), proteinuria and hypertension (41% each) as well as dysphonia (40%). The most common grade 3‐4 toxicities were asthenia (10%), neutropenia, reported in 15% of patients, while gastrointestinal toxicity and oral mucositis were each reported in 6.4% of patients, and hypertension in 3.8%.

**Table 4:** Summary of the most frequent all grade adverse events (≥20% of patients) and all grade 3/4 during combined aflibercept FOLFIRI‐treatment (N = 78)

	All grades		Grade 3/4	
	No.	%	No.	%
Asthenia	66	84.6	8	10.3
Diarrhea	50	64.1	5	6.4
Mucositis	45	57.7	5	6.4
Neutropenia	36	46.2	12	15.3
Proteinuria	32	41.0	0	0
Hypertension	32	41.0	3	3.8
Dysphonia	31	39.7	0	0
Anemia	21	26.9	1	1.3
Thrombocytopenia	16	20.5	2	2.6

## 4 DISCUSSION

This retrospective study confirms the efficacy of FOLFIRI‐aflibercept in a real‐world population and is, to our knowledge, one of the most mature studies in this setting in terms of follow‐up with a median follow‐up of almost a year. The importance of such studies is growing, with the absence of predictive response markers further prompting their development. In our study, the median OS of 12.0 months is slightly lower than that of the VELOUR study^4^ (13.5 months) while PFS was equivalent (6.8 months in our study vs 6.9 months in VELOUR), confirming the added value of FOLFIRI‐aflibercept in the second‐line setting in a real‐life population. The lower OS is likely due, at least in part, to the fact that in our study there was a higher rate of patients who did not receive further treatment compared to the VELOUR study (59% vs 40%). Furthermore, 30% of our patients were at least 70 years of age compared to only 5.4% in the VELOUR study, along with the fact there was a 53% rate of cardiovascular comorbidity in our study. On the other hand, our reported median PFS is longer than the 5.3 months reported in a Spanish named patient program (NPP) with aflibercept and FOLFIRI in an mCRC population with a comparable median age (64 years), but with a lower percentage of elderly patients (20% vs 30% in our study).^10^ Similarly, the ORR in our real‐life population (21.8%) matches those reported with FOLFIRI‐aflibercept in the clinical VELOUR study (19.8%), the retrospective NPP (19.7%), and the preliminary results from the noninterventional QoLiTrap study (22%).^4^, ^10^, ^11^ Response rates with FOLFIRI‐aflibercept are higher than those reported with other anti‐angiogenics in an equivalent setting, with an ORR of 13.4% with FOLFIRI‐ramucirumab and 5.4% with bevacizumab with oxaliplatin plus irinotecan‐based therapy.^12^, ^13^

Treatment exposure among our patients was also similar to that reported in the VELOUR study and higher than that in the safety report from the extended use ASQoP study,^14^ in which patients received a median of six cycles. A notable proportion of patients in our study (11.5%) received at least 25 cycles of FOLFIRI‐aflibercept. Toxicities linked to anti‐VEGF treatments (eg, hypertension, vascular events and proteinuria) were reported as expected in our study and incidences were similar to or lower than those reported for VELOUR, including hypertension (41% in both, all grades) including a remarkably lower incidence of grade 3/4 events in our study of 3.8% (all grade 3) vs 19% in VELOUR, proteinuria (62% vs 40%) and diarrhea (64% vs 69%, with grade 3/4 of 6.4% vs 19%). The lower rates of various toxicities in our study most likely reflect developments in the handling of aflibercept and adjustments made to FOLFIRI doses, given that just over half of the patients in our study had at least one dose reduction, with 15% having two or more. This is consistent with the NPP, in which 54% of patients had a dose reduction for FOLFIRI and 14% for aflibercept.^10^ Hematological toxicities were less common in our study compared to the VELOUR study, notably for neutropenia (46% vs 68% all grades, including grade 3/4 in 15% vs 37% in VELOUR), while anemia and thrombocytopenia were also less common in our study.

The real‐life population also demonstrated a trend of greater benefit in the subgroup of patients with metachronous metastases treated with FOLFIRI‐aflibercept than those with synchronous metastases. This may reflect that the fact that patients with metachronous disease typically undergo more frequent scans, so relapses are more likely to be detected in an asymptomatic population, with fewer metastatic sites and less bulky disease, favoring both better response and tolerance with chemotherapy. The extent of benefit of second‐line treatment is currently poorly understood, irrespective of the first‐line treatment received. No differences were identified for the extent of benefit with FOLFIRI‐aflibercept in this setting in terms of the type of prior first‐line therapy (ie, anti‐angiogenic vs anti‐EFGR or none). Nevertheless, this finding should be interpreted with caution given the low number of patients in each subgroup. Likewise, no differences were noted in the extent of the benefit according to *RAS* mutation status or surgery of the primary tumor.

The absence of validated clinical markers to predict the efficacy of aflibercept is a weakness for ensuring optimal treatment management with this agent. Various as‐yet unproven suggestions have been made that high or increasing levels of IL‐8 during treatment with FOLFIRI‐aflibercept are associated with lower PFS.^15^ Our study shows a strong correlation between the development of hypertension and treatment efficacy, with a 2.7‐fold reduction in the risk of progression. This development of an anti‐VEGF class toxicity as an efficacy marker has been reported with other anti‐angiogenic drugs.^16^, ^17^ Retrospective studies have reported an association between the development of grade 2/3 hypertension with bevacizumab in first‐line treatment of colon cancer in terms of response rate and PFS.^18^, ^19^ In addition, the development of hypertension during sunitinib treatment was associated with an improvement in efficacy in kidney cancer.^20^ Compared to other anti‐angiogenics, aflibercept is associated with a higher rate of hypertension.^21^ This is derived from its greater binding affinity to VEGF‐A, along with its binding to PIGF and VEGF‐B with independent pro‐angiogenic effects, and the longer half‐life of the fraction bound to the receptor compared to other anti‐angiogenics. Consistent with our study, Tahrini et al^22^ showed a correlation between the development of hypertension with aflibercept and increased survival in a phase II study. The study with sunitinib in kidney cancer also reported that patients who developed hypertension achieved better outcome.^20^

To date no there are no established biomarkers of efficacy for any anti‐angiogenic treatments, and this is the first study to associate the development of hypertension as an efficacy marker with second‐line anti‐angiogenic treatment. This finding needs to be corroborated in prospective studies. It is important to keep in mind that hypertension can be properly controlled without any need to prematurely discontinue treatment.

There is also growing interest in tumor laterality and its prognostic impact on mCRC. A meta‐analysis of 66 studies with nearly 1.5 million patients and a median follow‐up of 65 months reported a significantly higher likelihood of survival in patients with tumors located in the left colon.^23^ Our study is coherent with this and laterality is thus an important parameter to take into account when making treatment decisions.

## 5 CONCLUSION

Confirmation of clinically relevant efficacy in the real‐world population confirms FOLFIRI‐aflibercept as an attractive option in second‐line treatment for patients with mCRC following progression on prior oxaliplatin/irinotecan regimens. Tumoral *RAS* mutation status and administration of biological therapy (bevacizumab or anti‐EGFR) with first‐line chemotherapy did not significantly affect efficacy. Development of hypertension during treatment may represent a predictive response marker, a finding which merits investigation in prospective studies.

## CONFLICT OF INTEREST

None declared.
